# Support of adult urinary incontinence products: recommendations to assure safety and regulatory compliance through application of a risk assessment framework

**DOI:** 10.3389/frph.2023.1175627

**Published:** 2023-06-08

**Authors:** Edburga L. Krause, Anne M. Hattersley, Joan M. Abbinante-Nissen, Denise Gutshall, Kara E. Woeller

**Affiliations:** ^1^ Baby, Feminine and Family Care, Global Product Stewardship, The Procter & Gamble Company, Schwalbach, Germany; ^2^ Global Safety Surveillance and Analysis, The Procter & Gamble Company, Mason, OH, United States; ^3^ Baby, Feminine and Family Care, Global Product Stewardship, The Procter & Gamble Company, Cincinnati, OH, United States

**Keywords:** safety, incontinence (female), medical device, medical device regulation (MDR), biocompatibility, quality assurance, post-marketing surveillance, risk assessment

## Abstract

Urinary incontinence (UI) or involuntary loss of urine is a common chronic medical condition among women. It is estimated that 5%−70% of the population experiences incontinence with most studies suggesting 25%−45% of the population. Varying definitions of UI (e.g., stress, urgency, mixed) exist, and inconsistent symptom assessment tools, age, and gender can affect the estimate of incidence. Disposable Adult Incontinence products were first introduced into the market in the late 1970s and initially were used mostly in nursing homes and hospitals. However, during the 1980s, the market for incontinence products via retail outlets dramatically increased as awareness of the benefits of the products grew and stigma about their use declined. Today's products that manage urine loss have an extensive history and have evolved with time. Always products were introduced into the market in 2014 and are designed to meet the needs of women of all ages. Considered medical devices in some countries, regional regulations and global guidelines require clear planning, thorough assessment, and concise documentation of clinical safety. This manuscript will briefly review the regulatory landscape with a specific focus on European Union regulations. As previously published, the iterative, risk assessment framework used to assess the safety of Always incontinence products confirms that these products are compatible with skin and can be used safely. This manuscript will expand on the current literature highlighting additional steps that help assure the safety and compliance of the products from quality assurance programs through comprehensive post-market safety surveillance. Recommendations to help ensure several of the key regulatory requirements are met are outlined in the context of a risk assessment framework used to assure safety.

## 1. Introduction

Urinary incontinence (UI) is the loss of bladder control or involuntary loss of urine. In results from multiple countries, it is estimated that 5%−70% of the population experiences incontinence with most studies suggesting 25%−45% of the population. Urinary incontinence is more common in women and increases with age (1). There are two main types of urinary incontinence. Spontaneous urine loss that occurs with strenuous physical activity, a cough, sneeze or laugh is termed Stress Urinary Incontinence (SUI). Urine loss associated with a sudden compelling desire to void is termed Urgency Urinary Incontinence (UUI). Women who experience both types of symptoms are considered as having mixed urinary incontinence (2). Stress urinary incontinence is the most prevalent type occurring in approximately 50% of incontinent women. The next most common is mixed urinary incontinence at approximately 30% with urgency urinary incontinence at about 10% (3). Stress urinary incontinence is prevalent in women of all ages and is the most common type of urinary incontinence in women under 60 years of age (4). Mixed incontinence is most prevalent (55%) in women older than 60 years, with stress incontinence noted in only 25% and urgency incontinence noted in only 9% (5). Stress urinary incontinence can also occur in young nulliparous women considered to be elite athletes. Studies show that stress incontinence is more common in white women than black women (6).

Products to manage adult incontinence (AI) go back to at least 1550 BC where an Egyptian text on Papyrus discusses how to treat incontinence for both men and women and suggests vaginal inserts to provide compression for women. By the late 1800s, cloth diapers and safety pins were available for the masses. World War II drove inventors to start developing disposable diapers because women were busy working assembly lines and there was a shortage of cloth for diapers (7, 8). Pampers led the way for disposable baby diapers in 1961 by advancing the design of automated disposable diaper manufacturing machines. Procter & Gamble (P&G) took the success of Pampers diapers to the AI market and made the first AI brief (Attends) available to consumers in 1978. The scope of this market introduction of AI products was small and products were initially used mostly in nursing homes and hospitals. However, during the 1980s the market for incontinence products via retail outlets increased dramatically as awareness of the benefits of the products grew and stigma about their use declined. P&G re-entered the AI market with a full product line-up in 2014 with the launch of Always urinary incontinence products which included: liners, pads, and pull-on pants. Similar products under different P&G brand names are marketed around the world (e.g., Always, Whisper) and follow the regulatory compliance and safety strategies outlined in this publication. For the purposes of this paper, we will refer to the Always brand for ease of discussion.

**Figure 1:**
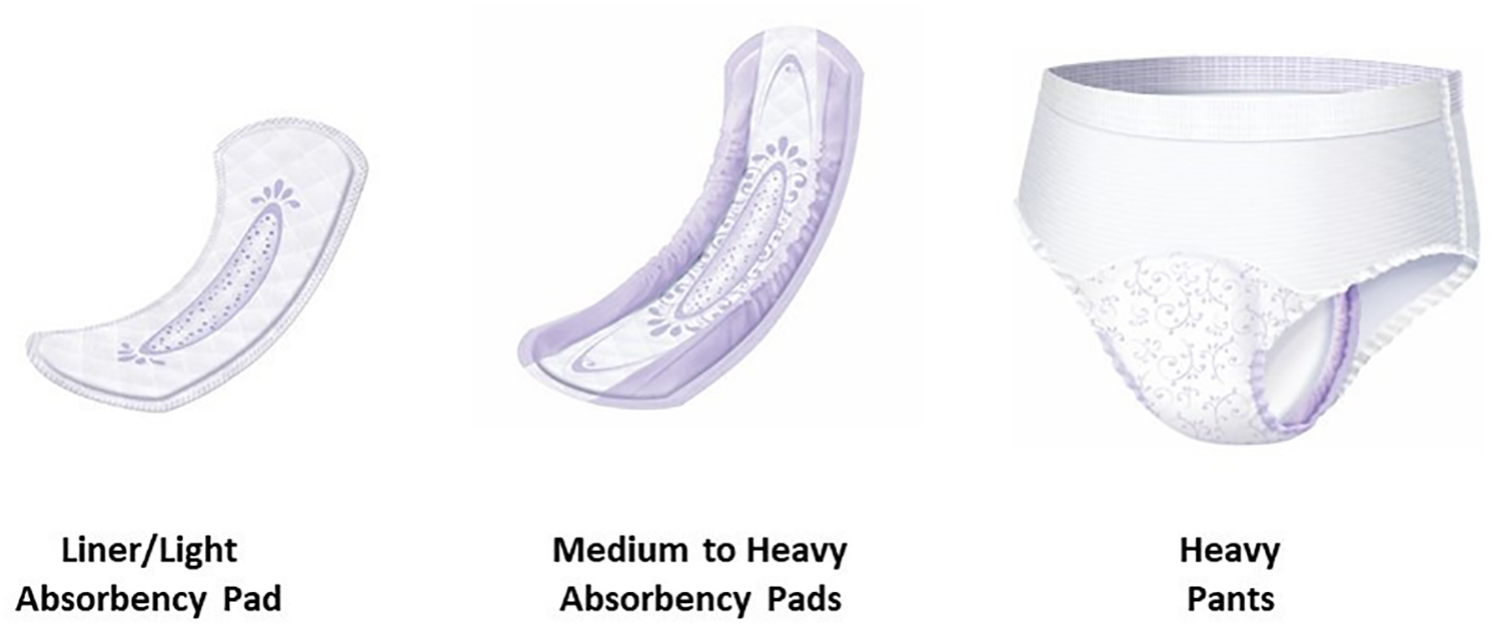
Adult incontinence product line-up. Figure provides an overview of the product line-up with regards to shape, size, and product type. Minor variations in color or product design will occur by region and product type.

The current Always AI product line-up is extensive and designed to meet the needs of all ages. For people with light to moderate incontinence, there are liners and pads; for people with heavy incontinence there are pads and pant products (Figure 1). For ease, the nomenclature throughout this publication will be referred to as: light, medium, and heavy. The pad product is applied to undergarments while the pants product can be pulled up and down like normal underwear. It is estimated that 90%–95% of AI pants used in developed countries are disposable. An average incontinent adult may be exposed to approximately a thousand disposable pants per year (9). These products are intended for an active female population suffering from light to heavy urinary incontinence, regardless of the type of incontinence.

The clinical risk associated with the use of these products is minimal. The scientific and clinical literature demonstrates that use of AI devices poses no unacceptable risk. Importantly, a clinical investigation performed on the Always AI pants product identified no serious adverse events and no withdrawals due to adverse events. The products were well tolerated by the subjects with favorable outcomes for both objective evaluations of skin health (erythema, trans epidermal water loss, red and pressure marking) and daily subjective reporting of comfort (10). Taken collectively, these data demonstrate that these AI products can be used safely by the intended population for the indication of urinary incontinence management. The articulated comprehensive approach, meets or exceeds United States (US) and international regulatory standards including regulations established in the European Union (EU) for medical devices. Safety surveillance data collected over the last 8 years since re-introduction into the market support these conclusions. Herein, we outline the EU regulations specific to the safe use of the device and provide an overview of the criteria needed to support regulatory compliance and safety support for these AI products.

## 2. Sections on assessment of policy/guidelines options and implications

Regulatory classification of AI products is often different from other assembled hygiene products, such as baby diapers and menstrual (sanitary) pads in several countries. The United States Food and Drug Administration (FDA) regulates AI products and menstrual pads as Class 1 medical devices subject to manufacturing controls and consumer complaint management. In the European Union AI products are also a Class 1 medical device while menstrual pads are considered articles. In Canada, absorbent AI products and menstrual pads are regulated as articles. In Japan, sanitary pads fall under the scope of the Pharmaceutical and Medical Device Agency, but AI products are a household product. However different the regulations are among geographies, there is still a fundamental expectation of a human health risk assessment of new products to ensure that these products are safe for the consumer. In this publication we provide insights with regards to the European Medical Device Regulation (MDR), which has undergone a significant extension of regulatory requirements versus the previous Directive and outline how we ensure, plan, and document the safety evaluation of our products. Recommendations on how to approach the various requirements are outlined throughout.

### 2.1. Regulatory framework in the EU

Always AI products are classified as class I medical devices in the EU, following the definition of medical devices in the Medical Device Regulation (11) to prevent, treat or compensate for a disease, injury or disability. With that, the Medical Device Regulation is applicable for this type of product, setting the regulatory framework for these products for the European Union market.

**Figure 2:**
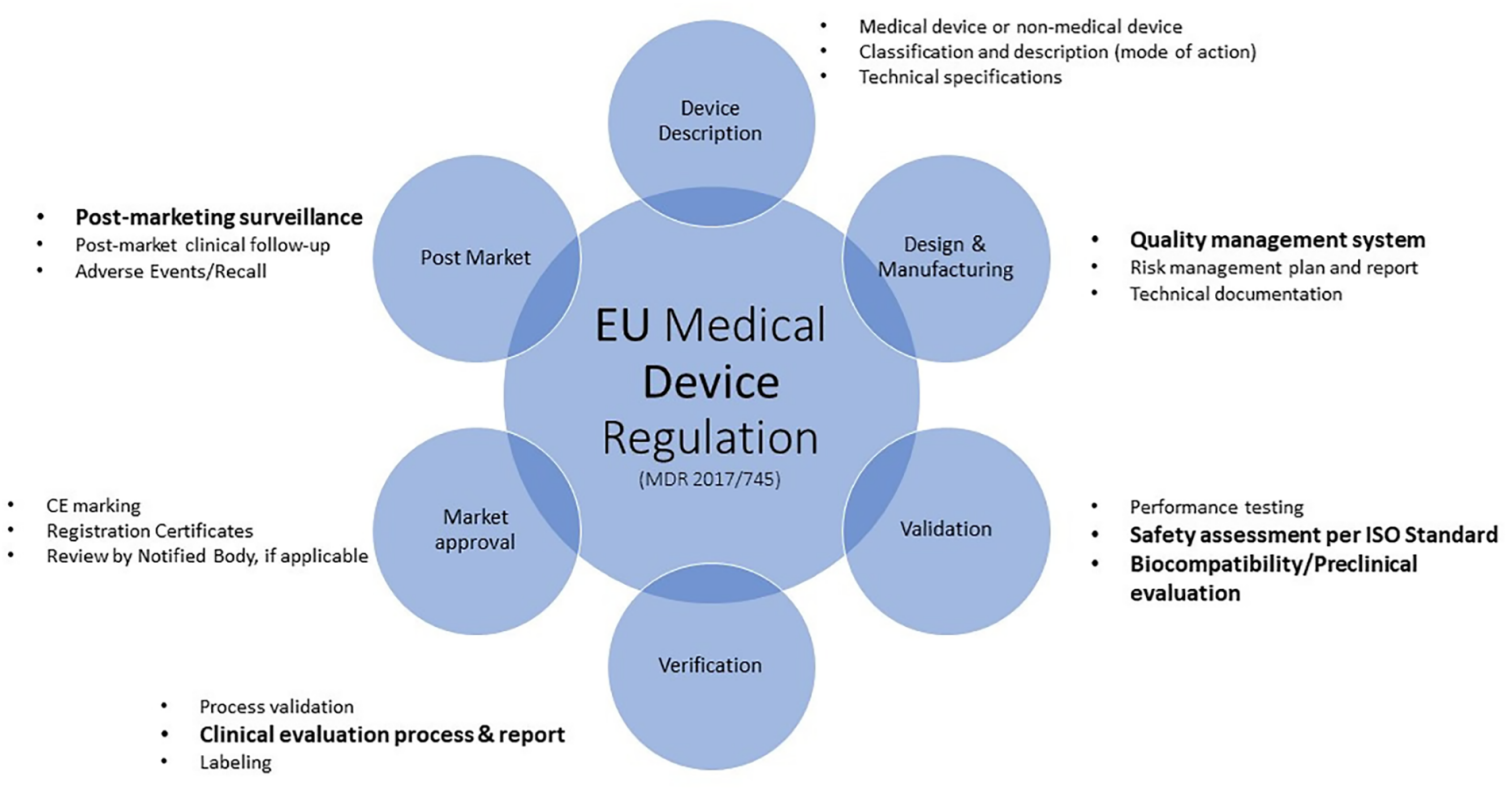
Overview of the EU medical device regulation requirements. The process described herein is not always linear and sequential as various aspects will feed into other elements. For example, clinical data from devices of similar composition discovered in the clinical evaluation process may provide support for the safe use of those materials. This information can be included in the material characterization information. Topics highlighted in **bold** text will be discussed.

The new Medical Devices Regulation (11), now fully applicable since 26 May 2021, brings EU legislation in-line with technical advances, changes in medical science and progress in law-making versus the previous Medical Device Directive (12) and outlines requirements to safely market Medical Devices, such as Safety evaluation, Quality Management and Post market surveillance (see Figure 2).

In addition to the requirements outlined in the MDR, there are guidance documents and International Organization for Standardization (ISO) standards which outline and present a common understanding of the regulation. The Medical Device Coordination Group (MDCG) endorses a range of guidance documents to assist stakeholders in applying Regulation (EU) 2017/745 to medical devices. The documents, which are not legally binding, present a common understanding of how the MDR should be applied in practice aiming at an effective and harmonized implementation of the legislation.

In order to demonstrate compliance of any marketed medical device in Europe, a Summary of Technical Documentation (STED) file is required. The STED file includes detailed information about the design, function, composition, use, claims, and clinical evaluation of a medical device. In general, for all classes of devices the Technical File must be reviewed by a Notified Body (NB), with the exception of Class I devices that are not provided sterile, do not have a measuring function and are not reusable surgical instruments. As AI products are classified as class 1 medical device (non-sterile, non-measuring), there is no review of the STED by a NB, prior to placing the device on the market. However, authorities can request to audit/see the STED file as part of an audit.

The safety assessment is a critical part in the evaluation of a medical device and is documented in the clinical evaluation report (CER) for the device. This CER is the documented outcome of the Clinical Evaluation Process (CEP) which is a systematic and planned process to continuously generate, collect, analyze, and assess the clinical data pertaining to a device in order to verify the safety and performance, including clinical benefits, of the device when used as intended as stated by the manufacturer (11).

The main goal of any CEP is to demonstrate the safety and performance of the medical device in clinical use which is an important part of the conformity assessment process to apply the CE (Conformité Européene) marking. The CEP consists of two phases: pre-market and post-market of the medical device. In the pre-market phase, the manufacturer of a medical device must prove that it meets the specified performance and is safe in order to legally market the product. This must be followed by clinical evaluation, which justifies certain risk management actions. During the second phase, post-market introduction, the clinical evaluation must be updated to capture ongoing monitoring with regard to clinical performance and safety. In particular, technical adaptations and optimizations of the product must be re-evaluated. This includes data from post-market surveillance of the product under evaluation as well as data from similar products across the industry available in public literature. When information from these data sources calls into question the conclusions reached during the pre-market assessment, these data are shared with the risk assessors who re-evaluate the safety of the medical device in light of this new information. When appropriate, the medical device is modified to reduce or eliminate the unexpected in-market experiences.

### 2.2. Clinical evaluation process

According to the guidelines, one should follow the following steps in the clinical evaluation:

**Step 1: Planning**
Outline the objective and structure of the clinical evaluation, classification of product development (known/new technology, new application), intended purpose, target user groups, methods and parameters used to determine the acceptability of the benefit-risk profile, and residual risks and side-effects (if applicable).
**Step 2: Identification**
Collect clinical data, consider equivalence, possible sources: scientific literature, clinical experience, clinical trial
**Step 3: Assessment**
Individual evaluation of data, assessment against established criteria, e.g., biocompatibility via the ISO standard 10993.
**Step 4: Analysis**
Overall evaluation of relevant data to assess whether evidence of medical device performance and safety is given. Conclusions on the benefits and safety of the product should be provided.
**Step 5: Report**
Logically structured report on the evaluation with justifications and documentation of the steps, documents all individual steps (Clinical Evaluation Report).

The CER must be actively updated, at least annually for medical devices with significant risk or devices that are not yet well established and every 2–5 years for devices not expected to be high risk. For AI products, which are a class 1 medical device, an update of the CER every 2–5 years is applicable. In practice for Always AI products, an approximate yearly review is conducted to include and document new literature and an up-to-date assessment of any new (post-market) data. In the following chapters we provide further information on the safety evaluation of Always Urinary Incontinence products, post-market surveillance data, as well as Quality Assurance obligations to ensure that the product is safe for the consumer. Recommendations for assessment of safety for a class I medical device with the type and contact duration (surface, medical devices with long term exposure to intact skin) similar to AI products are covered in this manuscript.

## 3. Actionable Recommendations

### 3.1. The safety assessment of adult incontinence products

The safety assessment process has been previously described for AI products (9, 13), as well as for other similar assembled, absorbent hygiene products (AHPs) such as baby diapers (14–16), and menstrual pads (17). Marsman et al. (9) discuss the principles governing Exposure-Based Risk Assessment (EBRA) while providing an example of the process for both systemic endpoints as well as local effects (i.e., sensitization) while Gutshall et al. (13) provide a summary of the clinical data demonstrating the favorable skin compatibility of these final, finished AI products. These manuscripts provide foundational support within public literature for the safe use of these products when used as intended and under foreseeable use conditions.

**Figure 3:**
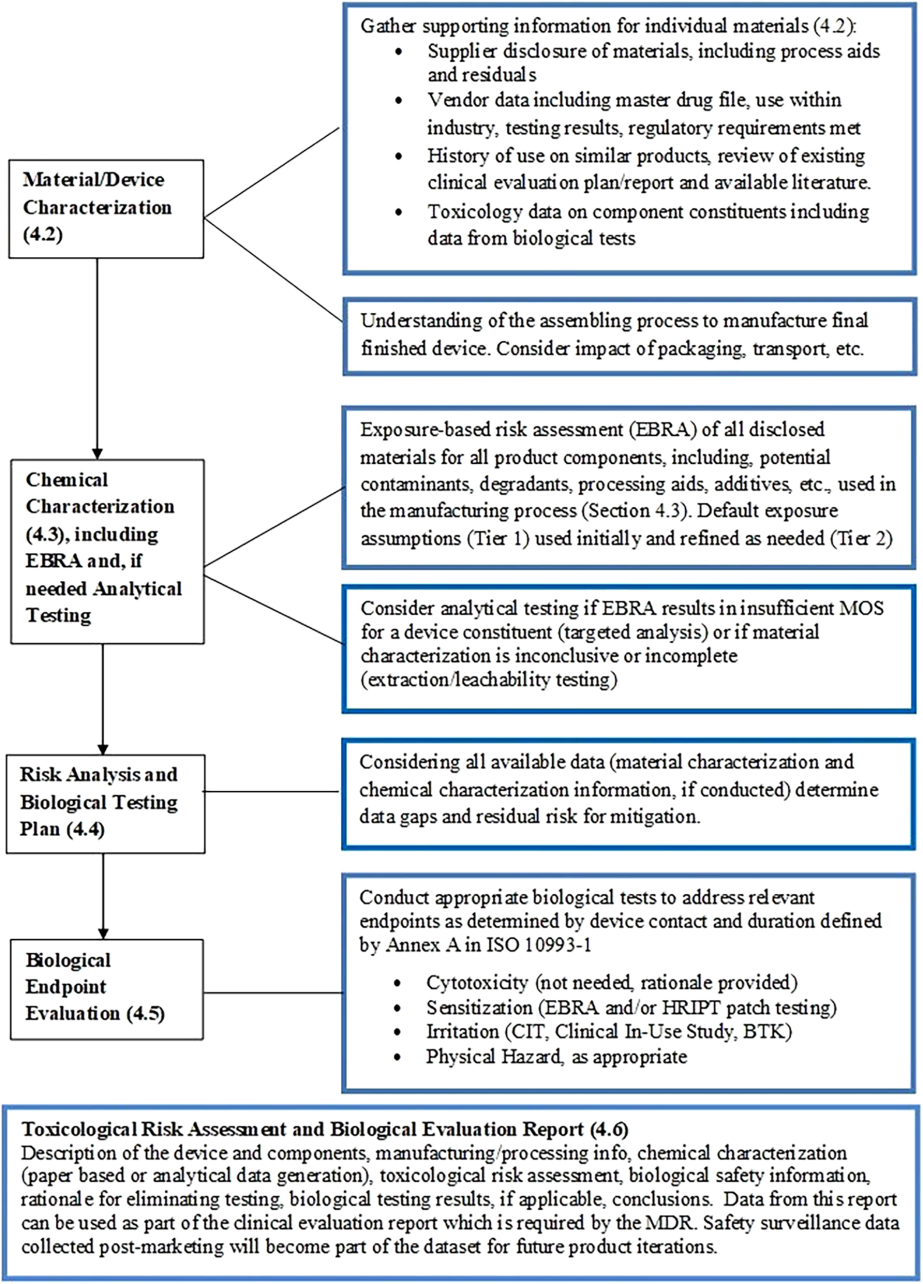
Biological evaluation plan for AI products according to the ISO 10993 standards.

While assuring safety is of utmost importance, equally important is ensuring that the product meets all local regulatory requirements. In many cases the work done to assure that the product is safe and compliant will overlap significantly. Often the safety assessment extends beyond those requirements defined by authorities. In the EU, medical device regulations require manufacturers to generate and document the general biological safety and performance requirements of the device (11). This plan, coined a ‘Biological Evaluation Plan’ and documented in the associated STED file, should contain a report on biocompatibility or a biological safety assessment report. The term biocompatibility as defined by ISO 10993-1 refers to “the appropriate response in a host to the situation of its use” (18). ISO 14971 (19) as well as the ISO 10993 series of standards provide a framework which guides device manufacturers in the assessment of medical device biological safety/biocompatibility. These standards are not meant to be prescriptive but rather a guide for evaluating the biological safety of a medical device within the risk management process as outlined in ISO 14971. While ISO 10993 is not the only standard that can be applied, the broad use and acceptance of the ISO 10993 series has led to its pre-eminence among the medical device standards. Following the steps outlined in these standards should provide sufficient evidence to adequately address chemical and biological risk for AI products (Figure 3).

### 3.2. Material characterization

Material selection is the cornerstone of the safety assessment process. Selection of appropriate materials that are compatible with human tissue relevant to the in-use situation should be part of the medical device design process. When possible, a history of use within the medical device industry and within the same product category is ideal. Once materials are selected, material characterization is a crucial first step in the biological evaluation of medical devices (18). Material characterization includes a thorough, data-based scientific understanding of each individual component in the assembled device. Material information can be obtained through review of the literature, vendor or in-house data or comparison with existing, marketed products where the manufacturing processes and formulations are known and the same as the medical device under evaluation (18). Obtaining compositional information, including alternative materials, processing aids, etc. from suppliers and a compilation of chemical constituents used in the individual product components as well as known and expected impurities, process aids, and additives that are used in the manufacturing of the device is key. Manufacturers must also understand how the manufacturing process (e.g., chemical interactions between product components, sterilization, packaging) may affect the suitability of these materials and how they interact with the body.

The Always AI products discussed in this publication have the conventional layered design described in EDANA's (European Disposables and Nonwovens Association) Absorbent Hygiene Product (AHP) components: AI liners, pads & pants (20). Practically speaking, these AI products are similar in their layers and composition, varying only in elements that deliver product function and performance (e.g., barrier leg cuffs). The products feature a permeable surface (topsheet), an acquisition layer (or secondary topsheet), an absorbent core and impermeable backing (backsheet). Product composition is detailed in Table 1. The topsheet is typically a polyethylene/polypropylene non-woven material designed to quickly transfer fluids (urine) to the layers below. The topsheet may contain a printed visual signal and/or a tint. The acquisition layer is composed of modified cellulose and polyester and is designed to facilitate the movement of liquid away from the skin to the absorbent core. The acquisition layer may be printed. The absorbent core consists of a superabsorbent polymer gel that is blended with cellulose and may be contained in a cellulose or polymer non-woven layer. The backsheet consists of a water-proof polyethylene film with panty-fastening adhesive for pads and liners. Most products contain perfume-like odor lock technology applied to the top of the absorbent core.

**Table 1:** Components of adult incontinence liners, pads and pants.

Component	Function	Raw material composition
Topsheet	Permeable surface cover that is soft to skin and allows fluid to penetrate	Non-woven fabric of polypropylene/ polyethylene fibers. May be printed and/or tinted.
Absorbent Core	Cellulose fibers quickly absorb fluid as part of the Always Discreet RapidDry Core while the Absorbent Gel Liquid-to-Gel Technology locks away fluid for a comfortable and dry wearing experience.	Cellulose, Rayon, Polyester, Polypropylene, Polyethylene, Absorbent Gel Material
OdorLock™ Technology	Perfume-like mixture that neutralizes and masks odor.	Perfume-like material
Backsheet	Prevent liquid from leaking out of the product	Polyethylene film
LeakGuard™ Leg Cuffs	Prevents fluid leakage out of the side of the pad or pant.	Polypropylene/polyethylene non-woven which may be tinted. May contain synthetic elastic fibers.
Adhesive	Attaches pad or liner to a panty or used to bind product components together	Polyaromatic/polyolefinic block copolymers, hydrocarbon resins, mineral oil
Colorants	To create a visual signal	Pigmented and polymeric dyes inks and polymer embedded colorants
Waistband	Soft cotton-like material on pant products with elastics allow the product to stay-in-place on the body.	Polypropylene/polyethylene non-woven with synthetic elastic fibers encased in the non-woven. Non-woven or elastic fibers may be tinted and/or printed.

The major components of materials that comprise the AI medical device are large molecular weight, inert polymers that carry a history of safe use and are extensively used throughout the absorbent hygiene industry. These polymeric materials have negligible systemic bioavailability and are therefore biologically inert. All product components in the final, finished medical device are also used in a number of other currently marketed absorbent products, such as baby diapers and feminine menstrual hygiene products, and are supported by a large body of toxicological data, with a long history of skin compatibility and safe use (13).

Each material used in the final, finished Always AI device is well characterized through supplier disclosure, including sub-suppliers, down to the chemical by Chemical Abstracts Service (CAS) # for material constituents and/or residual components. Both identity and quantity used are disclosed. The manufacturing process for AI products is an assembling process. Products are manufactured on a converting line and assembled by stacking product components and adhering these components together with adhesive. Importantly, the manufacturing process used to assemble these multi-layered products is controlled so that additives, process aids and residual chemicals are known and included as a part of the biological assessment.

**Figure 4:**
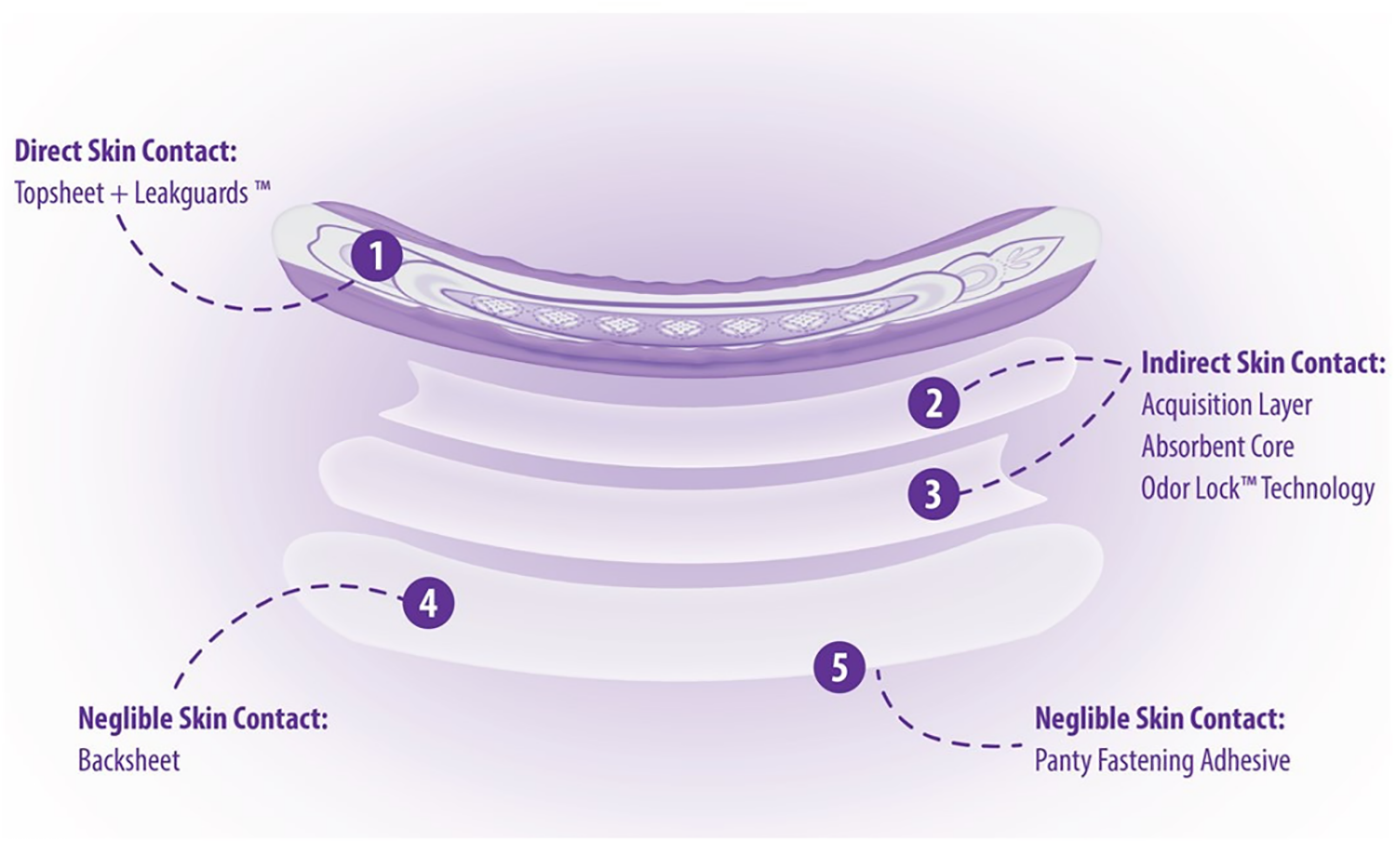
Adult incontinence components with respect to exposure using a pad example.

Part of material characterization is understanding how each material interacts with the body. Because Always AI products are assembled, layered products, estimating consumer exposure to the various material constituents will differ depending on the placement of the material within the product (Figure 4). Consumer exposure estimates can be derived from collected habits and practices (H&P), consumer data and an understanding of exposure dynamics of a 3-dimensional product. Modern disposable AI products are composed of mostly natural or synthetic large molecular weight polymeric materials (>99%) that are not bioavailable and are designed to pull wetness away from the consumer's skin. In assessing the individual incontinence product components for safety, one approach is to conduct a paper-based safety assessment based on supplier disclosures of starting materials used in making product components and applying conservative assumptions about how much of those disclosed constituents might actually come into contact with the consumer's skin based on the contact that product component has with the body during use. Three zones of exposure exist for these layered, assembled products: direct, indirect and negligible. Default assumptions for each zone of exposure have been derived (9, 20) (Table 2).

Direct Contact Materials (Zone 1): Incontinence product components, such as the polymeric topsheet with a printed visual signal, that are in direct contact with the skin during use (e.g., topsheet), can transfer constituents to the skin; solubilization in body fluids contained in the product is not required for constituent transfer. However, even with materials in direct contact with the skin, only a fraction of the total amount will transfer to the skin (9, 15). The assumption for topsheet constituent transfer is that any constituents in the topsheet would transfer to the same degree as a constituent that is intended to transfer to the skin (e.g., lotion). While AI products do not contain lotion, use of 7% transfer, which is based off lotion transfer data, is considered conservative for use in estimating exposure from direct skin contact materials based on previously cited data (9, 15).
Indirect Contact Materials (Zone 2): AI materials below the topsheet (e.g., acquisition layer, absorbent core, odor neutralizing technology) are not in direct contact with the skin but may contain constituents that can be carried to the surface via solubilization in urine or other body fluids and resurfacing to the skin. This phenomenon is coined rewet and is defined as the small amount of fluid migrating back from the product to the skin during the use of a product. Studies show that the default rewet factor AI products is approximately 1% depending on the product under assessment (9, 15). It is recommended to re-evaluate default rewet assumptions when estimating human exposure to AI product constituents especially if changes are made to the core absorbent materials.
Negligible Skin Contact (Zone 3): Product components such as the outer liner in AI pad products, that are not in contact with the skin, directly or indirectly, contribute negligibly, if at all, to the estimate of exposure. As an example, backsheet materials and adhesive constituents have no or negligible skin contact and no exposure from rewet. Data from historical experience, product integrity standards and analytical leachability evaluation support the assumption for no or negligible skin exposure to these materials (unpublished data).
This approach of using default assumptions as described above to estimate human exposure to direct and indirect product components is considered to be quite conservative and an exaggeration of what a consumer will be exposed to during normal product wear but an appropriate approach in the absence of chemical specific, analytical data (21). In many cases the actual exposure to constituents in the product will be negligible, especially in the case of materials that are not in direct contact with skin. These values and/or zones of exposure can be refined when a data-supported rationale is justified. Additionally, targeted analytical studies (tier 2) to augment supplier disclosure can be done to refine exposure estimates and are recommended when default assumptions (tier 1) yield an exposure that cannot be supported.

**Table 2:** Default exposure parameters to estimate consumer exposure to constituents in a medium adult incontinence product.

Parameter	Tier 1 value and units
Product component mass (RMW)	X grams/product
Concentration of chemical constituent in the product component (CP) as disclosed by supplier or measured via analytical means	Y%
Daily frequency of use (*n*)	3 pads/day
Transfer to Skin from product (RF)^a^	7% transfer
Rewet to Skin from zone 2 components (Rw)^b^	1% rewet
Dermal absorption factor (Ab); default to 100% unless data are available	100%
Exposure duration (ED); default to lifetime exposure	100%
Female Body weight (BW)	60 kg
Surface Area of material component (SA)	Z cm^2^
Conversion Factor for systemic endpoints (grams to milligrams conversion	1,000 mg/g
Conversion Factor for dermal endpoints (milligrams to micrograms conversion)	1,000 ug/mg

^a^ Assumes 100% of low molecular weight constituents with direct skin exposure transfer to the same degree (worst case).

^b^ Assumes 100% of low molecular weight constituents in the inner layers of the product are solubilized in absorbed fluid and transferred to the skin through rewet.

Default values used in Tier 1 are considered to be conservative, leading to an overestimate of exposure; default values can be refined in Tier 2 when product/chemical specific data are available.

### 3.3. Chemical characterization

Chemical characterization is defined by the ISO standards as the “process of obtaining chemical information, accomplished either by information gathering or by information generation, for example, by literature review or chemical testing” (18). While the ISO guidance suggests that this should be done, it does not provide specific guidance on how to use this information in the biological evaluation. One approach to chemical characterization is through literature review and compilation of comprehensive chemical constituent information from suppliers as described previously. Alternatively, chemical characterization via analytical testing, extraction or leachability testing, is another mechanism to chemically characterize the medical device. According to the ISO standard, chemical characterization via analytical means is most helpful in certain situations such as when: proprietary issues can be resolved, only one (or a small number of) constituent is changing, toxicity data are readily available or extraction/analytical studies are easily conducted. Chemical characterization information can be helpful in guiding the assessor in selecting the appropriate biological tests, conducting a more targeted assessment of only physiologically relevant extractables or in cases where the composition of materials is unknown to the manufacturer due to proprietary information. ISO 10993-18 (22) provides guidance on when chemical testing is appropriate and a framework for the types of tests that can be conducted. ISO guidelines suggest manufacturers characterize the medical device exposure first using a hypothetical worst case chemical release scenario. When exposure based on this scenario is deemed acceptable, no further chemical characterization work is needed. However, if the outcome based on worst case assumptions is not acceptable then follow-up extraction and/or leachability testing may be needed to refine the estimated exposure in a way that is more physiologically representative of the intended use of the product. While following a worst-case scenario is conservative in nature, it is recommended as a first approach in the tiered assessment strategy, saving on analytical resources and time, while still assuring safe use of the product.

For the Always AI products, the materials have been chemically characterized in their chemical composition via supplier disclosure. The composition is well known and tracked and documented within internal systems. This compilation of chemical constituents for each medical device component serves as the base of the assessment of risk and assumes a worst-case scenario in terms of exposure estimates (Section 3.4) Chemistry extraction or leachable studies have not been done for these AI products. However, analytical investigations exist on similar materials and products. Data generated from this work can be extrapolated to AI products given the similarity in product composition, commonality in manufacturing processes, and in-use extraction liquid (i.e., urine). The outcomes of these studies (not currently published) demonstrate the concept of minimal extractable components from products made of polymeric materials. They underscore the conservative nature of our approach to assess each supplier disclosed constituent rather than generate analytical data on the final finished device.

#### 3.3.1. Exposure-based risk assessment of device constituents

Once the composition information is compiled, the next step is to evaluate the disclosed constituents through an Exposure-Based Risk Assessment process. This process can be completed on chemicals obtained through supplier disclosure, which is the case for the AI products described herein and/or on chemicals identified and quantified via analytical extraction (e.g., chemical characterization) testing. Each constituent is subjected to evaluation for systemic toxicity as well as local endpoints using broadly recognized exposure-based risk assessment principles. The multi-step, tiered exposure-based risk assessment process follows the principles established by the United States National Academy of Sciences (23, 24), the World Health Organization (25) and the United States Environmental Protection Agency (26). Likewise, ISO 10993-17 (27) details a method for determining allowable limits for medical device chemicals. While the terminology used by these reviewing bodies may differ, the principles for risk assessment are similar. This interdisciplinary and iterative approach focuses on the scientific understanding and measurement of potential constituent hazards as well as potential constituent exposure, and ultimately the risk associated with them. Endpoints for evaluation include but are not limited to: genotoxicity, systemic toxicity (acute, subchronic, and chronic), developmental and reproductive toxicity, carcinogenicity as well as irritation, sensitization and physical hazards.

Briefly, the steps in quantitative exposure-based risk assessment include:

• Hazard Identification and Dose Response: identifying the nature of potential adverse effects based on the toxicological characteristics of the chemicals or materials in question,
• Exposure Characterization: quantifying the exposure of toxicological interest for specific routes by determining the magnitude, duration and frequency of exposure under conditions of consumer use, and
• Risk Characterization: comparing these quantitative estimates to safe benchmarks for which no significant risk of adverse effects exists with the incorporation of a margin of safety (uncertainty factor) where needed to extrapolate from experimental conditions.

Risk acceptability is based on having a margin of safety for each constituent's hazard to the dose established in the scientific literature that causes an adverse biological response and comparing it the estimated consumer exposure using conservative default exposure assumptions. The safety approach is iterative and can be refined with analytical data and product category-specific usage information.

The following example illustrates the exposure-based risk assessment process and the AI specific parameters using Irgafos as an example.

#### 3.3.2. Quantitative exposure-based risk assessment process example for systemic endpoints

The exposure-based safety assessment process described below is utilized for each product component constituent disclosed to P&G by a supplier. This assessment is specific to the product under evaluation utilizing appropriate consumer exposure assumptions generated from habits and practice knowledge to estimate exposure. In this example, a topsheet constituent with direct skin contact in a medium AI product is used and the focus is on systemic endpoints. An example of an assessment for local effects which uses a similar approach to that described below is provided in Marsman et al, 2017.

a. History of Use: Irgafos 168 also known as Phenol, 2,4-bis(1,1-dimethylethyl)-1,1′1″-phosphite is a secondary oxidant that is used in the processing of polypropylene, polycarbonate and polyester. It is widely used throughout industry to prevent discoloration or change in physical or mechanical properties. It is approved for use in food contact applications, children's toys and fabric and textile applications.
b. Hazard Data and Identification of Critical Health Endpoints: Hazard data on Irgafos is readily available within the public, medical and toxicological literature and within internal databases. The toxicology data suggests that this material is not genotoxic as evidenced in multiple *in vitro* and *in vivo* assays and not carcinogenetic as concluded in a 2-year dietary study in rats. The repeat dose toxicity potential was tested in multiple studies and in multiple species up to and including 90 days of repetitive daily exposure. In all cases, the highest dose tested was without effect and a No Observed Adverse Effect Level (NOAEL) was established at the highest dose tested (1,000 mg/kg bw/day) (28). Reproductive and development toxicity studies indicate that Irgafos 168 is not a reproductive or developmental toxicant in rats or rabbits up to and including a dose of 1,200 mg/kg bw/day. Studies to understand potential local effects such as irritation and sensitization show that Irgafos 168 is not irritating to the skin and the sensitization potential is low.
c. Determining Risk Value or Tolerable Intakes (TI): These hazard data demonstrate that Irgafos is a material of low toxicity potential, is well tolerated systemically when dosed repeatedly to laboratory animals, is not irritating and carries low sensitization potential. From these data, we can conclude that the appropriate Point of Departure (POD) value to utilize for systemic endpoints in our risk assessment is the repeat dose NOAEL of 1,000 mg/kg bw/day. The appropriate uncertainty factors (described below) are applied to the NOAEL of 1,000 mg/kg/day to derive the TI. This value (TI) will be used in comparison to the estimated exposure to derive the Margin of Safety (MOS).
  • Modifying/composite uncertainty Uncertainty Factor (MF) utilizes a factor of 1 to 10 ascribing to the areas of uncertainty in extrapolating from the study data to the human conditions, such as inter-individual variability, intra-individual variability and study duration:
    • UF1: Inter-individual variation among humans (10×)
    • UF2: Extrapolation of data derived in species other than humans (10×)
    • UF3: use of an oral subchronic NOAEL with residual levels of uncertainty associated with potential route to route differences (10×)
    • MF = 10  ×  10  ×  10 = 1,000
  • derivation of risk value/TI:
    T1 = NOAEL/MF
    T1 = 1000 mg/kg/day/1000
      TI=1mg/kg/day
d. Determining Exposure: Irgafos is a constituent in the topsheet of AI products, so it is considered a zone 1 (direct contact) constituent. Utilizing the exposure parameters and associated default values presented in Table 2, exposure estimates for Irgafos are:
    $$\begin{array}{rl} & \mathrm{Systemic}\;\mathrm{Exposure}\;\left(\mathrm{mg}/\mathrm{kg}/\mathrm{day}\right):\\ & \frac{\mathrm{RMW}*\mathrm{CP}*n*\mathrm{RF}*\mathrm{Ab}*\mathrm{ED}\;*\;1,000\;\mathrm{mg}/g}{\mathrm{BW}} \end{array}$$
    $$\begin{array}{rl} & \;\mathrm{Systemic}\;\mathrm{Exposure}\;(\mathrm{mg}/\mathrm{kg}/\mathrm{day})\;\;:\\ & \frac{\;*2.0\;\mathrm{grams}\;*\;0.00002*3\;\mathrm{pads}/\mathrm{day}\;*\;0.07*1*1*1,000\;\mathrm{mg}/g}{60\;\mathrm{kg}} \end{array}$$
    SystemicExposure(mg/kg/day):0.00014mg/kg/day
When hazard data suggest the potential for local effects (e.g., sensitization), a similar calculation can be done to estimate dermal exposure using the below calculation and expressing the exposure estimates in ug of chemical per cm2 surface area. No sensitization potential for Irgafos has been noted in multiple *in vitro* and *in vivo* studies, therefore, the calculation of dermal exposure is not needed.
    $$\begin{array}{rl} & \mathrm{Dermal}\;\mathrm{Exposure}\;\mathrm{mg}/\mathrm{kg}/\mathrm{day}:\;\\ & \frac{\mathrm{RMW}\;*\;\mathrm{CP}\;*\;n\;*\;\mathrm{RF}\;*\;\mathrm{Ab}\;*\;\mathrm{ED}\;*\;1,000\;\mathrm{nmg}/g\;*\;1000\;\mathrm{ug}/\mathrm{mg}}{\mathrm{SA}} \end{array}$$
e. Determining Margin of Safety (MOS): The MOS is determined by calculating the ratio of the TI compared with the level to which an adult human may be exposure using the calculation below:
  MOS: T1/Systemic Exposure
  MOS: 1 mg/kg/day/0.00014 mg/kg/day
    MOS:7143

A MOS value greater than 1 is typically judged by risk assessors and regulatory bodies to be unlikely to cause harm. If a margin greater than an acceptable level exists after considering uncertainties associated with both effects and exposure estimates, the risk may be considered low, and no further action is needed. Conversely, when the Tier 1 risk assessment suggests an unsupportable outcome, the default worst case assumptions can be reassessed based on specific scientific data available on both the constituent (e.g., dermal penetration) and the objective physiological exposure to the substance of interest under use conditions (e.g., duration and extent of exposure, relevant availability of the substance in the product under use conditions). The approach to risk assessment is iterative in nature and can result in multiple refinements of the initial conservative assumptions. Of course, if reasonable iterations do not result in a favorable margin of safety, the product components must be eliminated or modified before marketing, or mitigations put in place to reduce exposure.

This exposure-based risk assessment process is applied to each constituent.

### 3.4. Risk analysis for data gaps and potential for biological testing

Per the ISO guidelines, if data gaps identified through the material and chemical characterization process cannot be filled by available information or if there is reason to believe that the chemical composition of the final finished device may not reflect the chemical composition of the individual device components in a meaningful way (e.g., active chemistry), then subsequent biocompatibility testing for the applicable endpoints may be required. These studies may also be done as a weight of evidence to assure safety or for endpoints that cannot be assessed through an exposure-based risk assessment (e.g., irritation). However, according to the ISO standard, “if the combination of all materials, chemicals, and processes has an established history of safe use in the intended application, and the physical properties have not changed, then it is possible that further characterization and additional data sets (e.g., chemical analysis of extracts or biological testing) will not be necessary.” (18). Annex A of ISO 10993-1 provides a listing of endpoints relevant for various types and durations of exposure. As one of the goals of the ISO 10993 approach to biocompatibility is to reduce or eliminate unneeded animal testing, it is emphasized that simply planning to conduct testing against all of the aspects of biocompatibility identified in Annex A is unacceptable and does not meet the requirements of ISO 14971 or ISO 10993-1. Therefore, it is recommended that focus remain on the clinically relevant endpoints and to consider *in vitro* or *ex vivo* studies when possible.

Current Always AI products are single-use, disposable products that can worn daily by active adult women. Cumulative exposure can amount to daily exposure for several weeks, months or years. These AI products are not intended for institutional use which may have different wear patterns and frequencies, particularly when users require caregiver assistance to change their pads or pants. Always AI products are considered ‘surface, medical devices with long term exposure to intact skin’ based on the rationale laid out in 3.4.1 and 3.4.2.

#### 3.4.1. Type of contact

During use, these devices contact a women's genital region and/or abdomen and buttocks. The majority of this contact is to intact skin in the case of pants (abdomen and buttocks); the remaining contact for pants as well as for pads is with the female external genitalia which is highly similar to intact skin in terms of morphology and physiology. The mons pubis, the labia, the clitoris, and the perineum, have a keratinized, stratified squamous structure with sweat glands, sebaceous glands and hair follicles like skin at other anatomical sites; only the vulvar vestibule is non-keratinized, resembling that of the vagina and buccal mucosa (29).

**Figure 5:**
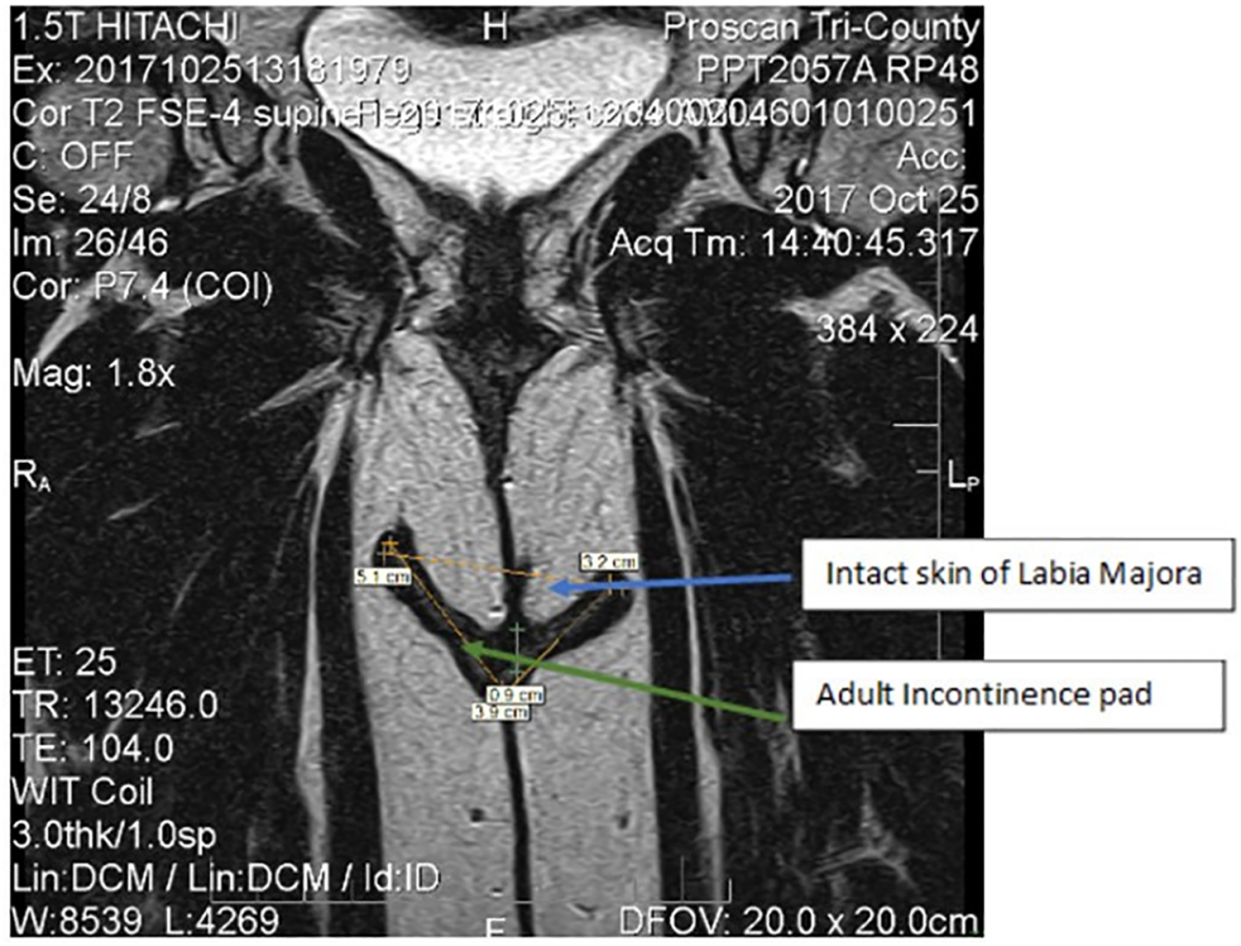
Frontal plan images via magnetic resonance imaging of female anatomy of the lower urogynecological region. The yellow line shows the orientation of the pad in contact with external structures of the pad (green arrow). The blue arrow shows the structures of the labia majora.

The labia majora form the folds that cover and protect the more internal structures of the vulva, such as the labia minora, clitoris, urinary orifice and vaginal opening (30). Figure 5 shows a coronal or frontal plane image taken via magnetic resonance imaging (MRI). Images, obtained at Proscan Tri-County, OH under Institutional Review Board (IRB) approved protocol [informed consent documents sent to Advarra IRB Columbia, MD, USA for review; study was conducted in compliance with the applicable Federal Guidelines for Good Clinical Practice (31), were analyzed using RadiAnt DICOM viewer) technology and show how the AI pad interacts with these structures of the body during use. The pad (yellow line) sits squarely between the upper thigh adipose tissue and the labia majora. The pad does not alter the normal position of the labia majora, allowing it to retain its function as a protective barrier and minimizing contact between the pad and the vulva vestibule and other internal structures/anatomy. Therefore, the majority of the contact the incontinence products have with the body is to intact skin (e.g., abdomen and buttocks, external genitalia), with negligible, incidental contact with mucosal tissue.

#### 3.4.2. Duration of contact

Based on understanding of product usage as well as the prevalence of urinary incontinence in adult women, these products may be used for a brief period after giving birth when women first experience urinary incontinence through later years during menopause and thereafter when urgency incontinence is more prevalent. Given this potential use pattern, the repeated long-term use of this product can exceed 30 days of cumulative use and as such this device is categorized as a long-term device.

#### 3.4.3. Relevant biological endpoints based on type and duration of contact

Given the above, these products are considered surface medical devices with cumulative, long-term contact with intact skin. Even though the exposure estimates conservatively assume daily, continuous use of these products as a default, it is notable, that not all women will wear these products continuously but rather for activities outside the home or overnight. As such, ISO-10993-1, Annex A indicates the following endpoints are to be evaluated in a biological risk assessment: physical and/or chemical information, cytotoxicity, sensitization, and irritation (Table 3). This is not a prescriptive measure for testing, but rather a guide for evaluating biological safety within a risk management process.

**Table 3:** Excerpt from ISO 10993-1, annex A: endpoints to be addressed in a biological risk assessment.

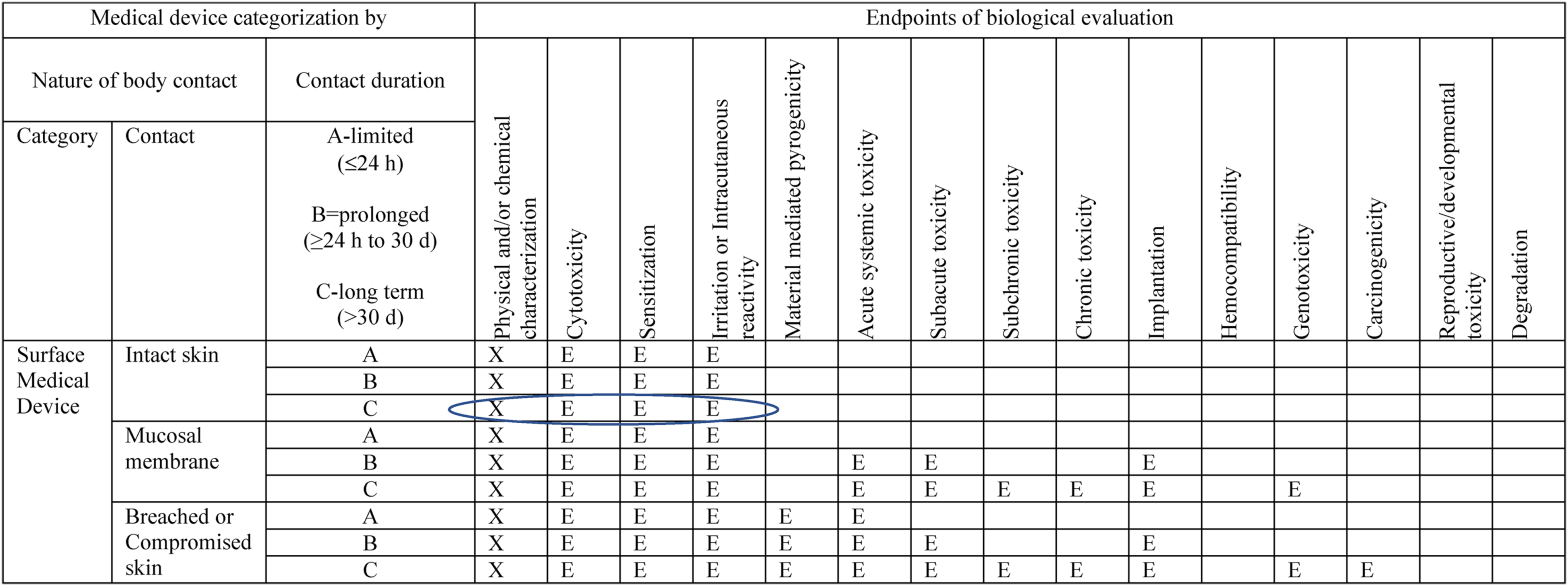

“X” means prerequisite information needed for a risk assessment.

“E” means endpoints to be evaluated in the risk assessment (either through the use of existing data, additional endpoint-specific testing, or a rationale for why assessment of the endpoint does not require an additional data set).

Blue circle denotes relevant endpoints for evaluation for a medical device which has cumulative contact with intact skin for >30 days in duration (e.g., long term duration).

The materials used to manufacture the products are well-known materials with a well-established history of biocompatibility and have been used in approved medical devices throughout the World for many years (Section 3.2). During the extensive literature review of the materials that was conducted as part of the material/chemical characterization process, there were no studies or reports found indicating that the materials posed a significant risk of cytotoxicity, systemic (acute, sub-chronic and chronic) toxicity, reaction (i.e., irritation), genotoxicity, carcinogenicity, reproductive/developmental toxicity, or adverse reaction following implantation. Likewise, the EU Clinical Evaluation Report concluded that the risk associated with the product is very minimal compared to the benefits. Importantly, Always AI products of very similar composition have been on the market and used safely by women since market introduction in the US and EU following Class I medical device requirements and EU Medical Device Directive, respectively, in addition to the history of safe use by competitive products.

Given the nature and duration of contact of this product with the body, as well as the demonstratable safe history of use of the components and final current disposable, AI product line-up, no biological testing is needed for the endpoints of systemic toxicity (acute, subchronic, chronic), implantation, reproductive and developmental toxicity, genotoxicity, and carcinogenicity (Table 3). However, as outlined in previous publications, P&G goes beyond the required endpoints of cytotoxicity, sensitization and irritation, and considers all toxicological endpoints for all chemical constituents in the individual product components that go into the AI medical devices we market.

### 3.5. Biological endpoint evaluation

Based on the type and duration of contact with the human body, cytotoxicity, sensitization and irritation are the relevant endpoints that must be addressed. As stated previously these endpoints are assessed first through material and chemical characterization to determine if a hazard exists for a given chemical constituent. In the case of sensitization this includes a quantitative EBRA and comparison to an appropriate reference value when a chemical with sensitization potential is identified. However, if further evaluation was deemed necessary, testing should be done on the modified device material or on the final finished device since some elements could impact final product biocompatibility. Testing for these biological endpoints is considered when design changes occur and/or products are modified. The impact of the change determines whether or not testing is appropriate. The following paragraphs provide a brief overview and recommendation for the tests that can be done in the evaluation of the relevant endpoints and summarizes the data collected from product introduction to present day. The final decision on how to test should also take this into consideration.

#### 3.5.1. Cytotoxicity

These tests determine cell death (e.g., cell lysis), the inhibition of cell growth, colony formation, and other effects on cells. Cytotoxicity tests are often seen as screening assays with a high degree of false positive and false negative results (32). Often follow-up studies are recommended to understand unfavorable data generated in these screening studies: a cytotoxic result “is primarily an indication of the potential for *in vivo* toxicity and the device cannot necessarily be determined to be unsuitable for a given clinical application based solely on cytotoxicity data” (33). The AI product materials are well characterized and suggest minimal likelihood of cytotoxicity potential. This is corroborated through clinical testing which shows the product to be compatible with skin (Section 3.5.3). No cytotoxicity studies were performed on the AI products. This assay is deemed unnecessary given the information gathered in the chemical/material characterization process.

#### 3.5.2. Sensitization

A Human Repeated Insult Patch Test (HRIPT) is a modified predictive patch study that can detect weak sensitizers that require multiple applications to induce a cell-mediated (Type IV) immune response sufficient to cause an allergic reaction. Over a course of 6 weeks nine patches containing the test material are applied on alternating days for three weeks to subjects who meet protocol described inclusion/exclusion criteria. Patches are worn for 24 h and then removed. Sites are graded prior to each patch application (24 h after removal during the week and 48 h after removal over the weekend). A 2-week rest period follows the last grading interval. During the 6th week, two patches are applied, one to the original site and one to a previously unexposed site, left in place for 24 h and then removed for grading (∼48 and 72 h after patch application). Nine different product iterations were evaluated in Human Repeat Insult Patch Tests (four of these products were tested in a self-assessed skin adult population) in over 1,000 subjects. None of them have shown the potential for sensitization. The assessment for sensitization includes a quantitative EBRA in conjunction with any testing (e.g., HRIPT) as deemed most appropriate for the device and country of distribution.

#### 3.5.3. Irritation

This endpoint is addressed through cumulative irritation patch testing as well as a Clinical Safety-in-Use study as summarized below.

• Cumulative Irritation Patch Testing: is designed to assess the irritation potential of a test product on the skin of human test subjects after a period of time and to detect weak irritants, which require multiple applications to cause a skin reaction. Irritancy reactions are due to direct damage to the epidermal cells; no immunologic (allergic) mechanism is involved. This procedure may detect so-called “fatiguing substances” which are mild irritants that cause more strongly positive reactions with successive, multiple exposures. Typically, between 15 and 35 subjects are tested in clinical irritation studies. The test product is cut into a 2  ×  2 cm^2^ patch, and wetted with saline or other appropriate vehicle, and applied to randomized patch sites to minimize possible site variation. The patch sites are evaluated every day for skin irritation and identical patches are applied to the same sites for consecutive days. This procedure is repeated daily for a total of 4–21 days. Positive, negative, and vehicle controls are usually included. Fifty AI product iterations were tested in cumulative irritation patch studies in more than 400 subjects. All products demonstrated skin compatibility similar to the negative control.
• Clinical Safety-In-Use Study: A multi-center, randomized, examiner-blinded, 2-week parallel single product use test was conducted in women who experience incontinence approximately 20% of whom were diabetic. Two products were tested: P&G pant and a competitor's product, Depends for Women. Of the 122 subjects 22 were diabetic and 88 were post-menopausal. Under the conditions of this study, there were no statistically significant differences in overall change from baseline for visual grading and trans-epidermal loss between the experimental product and the marketed reference product for all subjects. Changes from baseline for skin erythema and skin marking were generally small for both products for all subjects as well as for both diabetics and non-diabetics. There were no serious adverse events and no withdrawals due to AEs. Overall comfort assessments were favorable (10).

#### 3.5.4. Other remaining endpoints for consideration

Each of the endpoints listed in Annex A, including but not limited to acute through chronic, implantation, genotoxicity, carcinogenicity, reproductive/developmental toxicity was considered as part of the biological risk assessment. This is common practice for all P&G marketed products and is recommended as part of the chemical characterization process even though ISO does not require it given the type and duration of contact (18, Annex A). Based on the available literature data, the assessment of chemicals disclosed by the supplier for each component, and the lack of transformation during manufacturing as well as the clinical and in-market history (described in the CER) and current post-marketing safety surveillance data (Section 4), there is no need for additional biological testing for these endpoints. It is recommended that re-evaluation and potentially new testing, is considered if new scientific data indicate new concerns for current product components, or if signals occur from post-marketing surveillance or CER summaries.

Given that AI products are 3-dimensional, an evaluation of Physical Hazards (PH) is an important part of a finished product safety assessment and risk management plan. A physical hazard assessment is an evaluation of objective and perceived safety of a consumer product under intended and reasonably foreseeable use conditions. A physical hazard associated with a consumer product can be defined as any part of the product or its associated package that has a potential to cause injury leading to hazards pertaining to but not limited to choking/aspiration, cuts or skin abrasions, pinching or entrapment of body parts, suffocation, strangulation, etc. The specific hazards of relevance to a given consumer product will depend on the final product design/materials as well as the intended user (e.g., adult vs. child). Specific regulatory requirements for physical hazards of disposable AI products such as those described in this paper do not exist. However, it is recommended that the manufacturer evaluate the potential for these hazards as part of the research and development process and through post market surveillance data. Where appropriate, steps can be taken to address findings through product design changes and/or product labeling. If needed, the manufacturer can partner with external vendors who specialize in this area to provide perspective and/or testing.

### 3.6. Toxicological risk assessment documentation and biological evaluation report

The Biological Evaluation Report should provide documentation to support the biocompatibility/biological safety of the device. The ISO standards provide a listing of the appropriate content. Briefly, the report should include a general description of the medical device, quantitative information on the materials, a description of processing conditions that could introduce manufacturing contaminants, a review of the available toxicity and prior use data, reports of biological tests, assessment of the data and a statement confirming the risk analysis and risk controls have been completed.

For the AI products, the biological evaluation report concludes a favorable MOS for all disclosed constituents and negligible impact from the manufacturing process. The completed biological tests for sensitization and irritation provide important evidence that there is negligible concern for local adverse effects at the tissue-device interface when in contact with intact skin. The Biological Evaluation Report concludes that the device is safe for its intended use.

## 4. Post-market surveillance

In line with regulatory compliance requirements for medical devices (34), P&G maintains a post-marketing (passive) surveillance system to collect and assess adverse health events (adverse events, AEs) ascertained from comments that consumers, their relatives, or other individuals provide voluntarily about the company's products. This surveillance system has been described previously (35). Briefly, reports of consumer comments are collected via various communication methods (e.g., phone calls, e-mail, and company-sponsored social media) and maintained in a global database. An AE is defined by world-wide regulatory agencies as any undesirable effect on an individual's health and/or well-being associated with the use, misuse/overuse (intentional or not), off-label use of a product, or accidental/occupational exposure, whether or not it is considered product related (a causal relationship with the use of the product may not exist). Consumer comments and complaints may describe one or more AEs. The AEs are categorized using the Medical Dictionary for Regulatory Activities (MedDRA, www.meddra.org) terminology and mapped to Preferred Terms (PTs). Product safety is assessed frequently by reviewing and summarizing the AE data.

Here, we report on voluntary reports of Always AI products AEs obtained from the post-marketing surveillance system to augment our understanding of product safety. P& G maintains a standardized database which includes AE data for these Always AI products since market introduction.

We queried the surveillance database for cases coded to Always Light AI (LAI), Always Medium AI (MAI), and Always Heavy AI (HAI) products from August 1, 2014, to December 31, 2022. Descriptive analyses (i.e., frequencies) were conducted on case counts, demographic characteristics, and PTs for each AI segment (light, medium, heavy) separately. We assessed safety by examining the frequency (i.e., most commonly reported) and type of PTs. We also estimated reporting rates of AE cases based on shipment data (AE cases per one million Always AI products shipped). Our descriptive analysis used existing and anonymized data; an Institutional Review Board's approval was therefore not required.

### 4.1. Always Light Adult Incontinence (LAI)

In total, 1,492 Always LAI AE cases were reported (Table 4). An increase in cases occurred from 2021 (*N* = 147) to 2022 (*N* = 237) due to consumer comments indicating a “perfume”, “fragrance”, or “scent” issue with the product. The complaints referred to a change in formulation of the Odor Neutralizing Material (ONM) used to mask urine scent in AI products. From August 1, 2014, through December 31, 2022, the overall reporting rate was 0.3 AE cases per one million Always LAI products shipped. For cases with age group known, 23.7% (*N* = 353) were reported in the older adult (defined as 65 years of age and older) and 21.5% (*N* = 320) in adults (Table 5). Telephone (*N* = 813, 54.5%) and e-mail (*N* = 523, 35.1%) were the preferred reporting methods. The five most commonly reported PTs across all age groups were genital discomfort (*N* = 284, 19.0%), pruritus genital (*N* = 238, 16.0%), hypersensitivity (*N* = 176, 11.8%), genital rash (*N* = 146, 9.8%), and skin irritation (*N* = 142, 9.5%) (Table 6). Genital discomfort included complaints of irritation or discomfort in connection to LAI products. Hypersensitivity events indicated that the consumer reported an allergy or allergic reaction. The PTs for Always LAI correspond to expected AEs from LAI products that cover and contact the genital areas and surrounding skin. Each LAI case reported an average of 2.7 AEs (median = 2).

**Table 4:** Frequency and shipment-adjusted reporting rates of reported adverse events associated with Always adult incontinence products, by segment and year, 2014–2022.

Segment	Cases^a^ and Rates	2014^b^	2015	2016	2017	2018	2019	2020	2021	2022	Total
Always Light Adult Incontinence (LAI)	Adverse Event Cases (*N*)	91	187	140	149	163	169	209	147	237	1,492
	Reporting rate^c^	2.4	1.0	0.3	0.2	0.2	0.2	0.2	0.2	0.2	0.3
Always Medium Adult Incontinence (MAI)	Adverse Event Cases (*N*)	189	304	250	268	210	218	280	250	631	2,600
	Reporting rate^c^	8.7	2.9	0.7	0.5	0.3	0.3	0.3	0.2	0.5	0.4
Always Heavy Adult Incontinence (HAI)	Adverse Event Cases (*N*)	77	217	188	255	246	305	243	229	407	2,167
	Reporting rate^c^	2.7	3.6	1.4	1.4	1.1	1.1	0.8	0.6	1.1	1.1

^a^ Cases may appear in more than one segment if the consumer reported multiple products across segments.

^b^ Data from August 1, 2014, through December 31, 2014.

^c^ Reporting rate = AE cases per one million Always adult incontinence products shipped.

Reporting rates are often high once a product is introduced into market; reporting rates will generally stabilize over time as more products are used by consumers.

**Table 5:** Characteristics of reported adverse event cases^a^ associated with Always adult incontinence products, by segment, August 1, 2014—December 31, 2022.

Characteristic	Always Light Adult Incontinence (LAI)		Always Medium Adult Incontinence (MAI)		Always Heavy Adult Incontinence (HAI)	
	*N* = 1,492		*N* = 2,600		*N* = 2,167	
	*N*	%	*N*	%	*N*	%
**Gender**						
Female	1,460	97.9	2,536	97.5	2,084	96.2
Male	3	0.2	12	0.5	13	0.6
Unknown	29	1.9	52	2.0	70	3.2
**Age group**						
Child (<18 years)	1	0.1	4	0.2	7	0.3
Adult (18–64 years)	320	21.5	516	19.9	333	15.4
Older Adults (65 years +)	353	23.7	925	35.6	863	39.8
Unknown	818	54.8	1,155	44.4	964	44.5
**Region**						
Asia	28	1.9	20	0.8	17	0.8
EIMEA^b^	377	25.3	303	11.7	173	8.0
Latin America	0	0	0	0	1	0.1
North America	1,087	72.9	2,277	87.6	1,976	91.2
**Quarter (Seasonality)**						
First (January—March)	371	24.9	599	23.0	562	25.9
Second (April—June)	385	25.8	737	28.4	537	24.8
Third (July—September)	362	24.3	657	25.3	522	24.1
Fourth (October—December)	374	25.1	607	23.4	546	25.2
**Primary Reporting Source^c^**						
E-mail	523	35.1	693	26.7	453	20.9
Letter	30	2.0	42	1.6	29	1.3
Phone	813	54.5	1,660	63.9	1,463	67.5
Reviews	40	2.7	98	3.8	103	4.8
Social Media (company-sponsored)	61	4.1	76	2.9	71	3.3
Web Site (company-sponsored)	10	0.7	23	0.9	43	2.0
Unknown	14	0.9	8	0.3	4	0.2
Other	1	0.1	0	0	1	0.1

^a^ Cases may appear in more than one segment if the consumer reported multiple products across segments.

^b^ EIMEA includes Europe, India, the Middle East, and Africa.

^c^ The primary reporting source was provided when multiple sources were reported. Reviews = Report from ratings/reviews site. Other = Known report form, not fitting any other category listed.

Percentages may not add to 100.0 due to rounding.

**Table 6:** Frequently reported (top 15) adverse events associated with Always adult incontinence products from post-marketing surveillance, by segment, August 1, 2014—December 31, 2022.

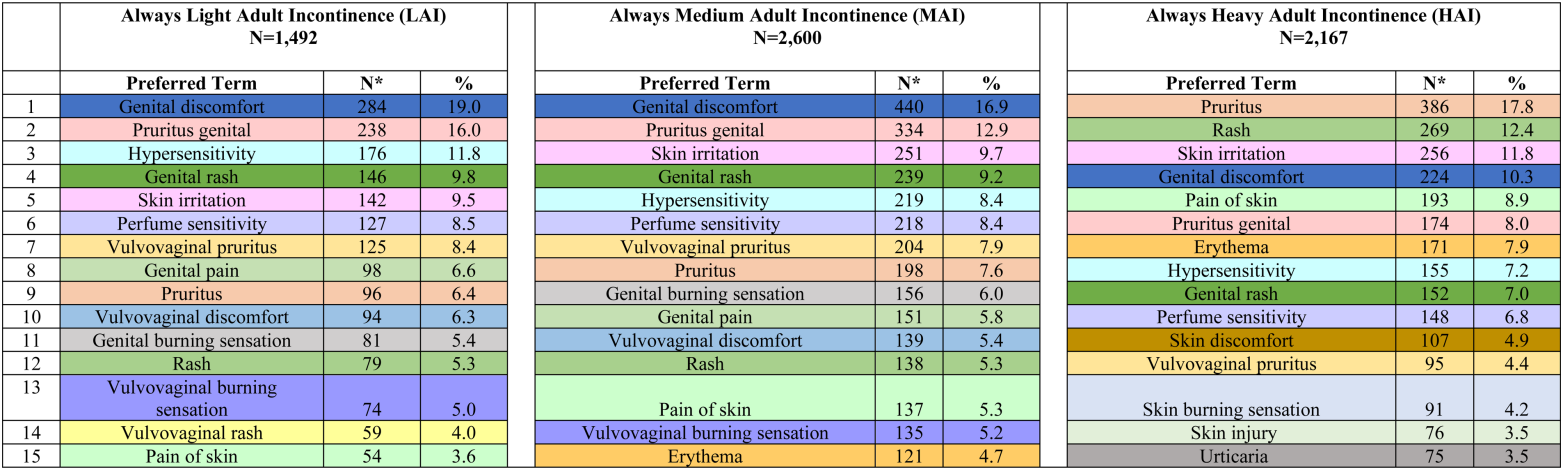

^a^ Each case may have one or more preferred terms. Cases may appear in more than one segment if the consumer reported multiple products across segments.

Colors were randomly selected and have no impact on the preferred term, and the same preferred terms may not be present in every segment.

### 4.2. Always Medium Adult Incontinence (MAI)

In total, 2,600 Always medium AI AE cases were reported (Table 4). Cases increased more than 2.5-fold from 2021 (*N* = 250) to 2022 (*N* = 631) due to consumer complaints about “perfume” issues with the products (discussed above in LAI). From August 1, 2014, through December 31, 2022, the overall reporting rate was 0.4 AE cases per one million Always MAI products shipped. The reporting rate, “normalized” to shipments, remained low in 2022 despite the increase in cases (0.2 in 2021 vs. 0.5 in 2022). For cases with age group known, 35.6% (*N* = 925) were reported in the older adult and 19.9% (*N* = 516) in adults (Table 5). Telephone (*N* = 1,660, 63.9%) and e-mail (*N* = 693, 26.7%) were the preferred reporting methods. The five most commonly reported PTs across all age groups were genital discomfort (*N* = 440, 16.9%), pruritus genital (*N* = 334, 12.9%), skin irritation (*N* = 251, 9.7%), genital rash (*N* = 239, 9.2%), and hypersensitivity (*N* = 219, 8.4%) (Table 6). Genital rash included complaints of a rash or unspecified breakout in the genital area. The PTs for Always MAI correspond to expected AEs from MAI products that cover and contact the genital area and surrounding skin. Each MAI case reported an average of 2.8 AEs (median = 2).

### 4.3. Always Heavy Adult Incontinence (HAI)

In total, 2,167 Always HAI AE cases were reported (Table 4). An increase in cases occurred in 2022 (*N* = 407 vs. *N* = 229 in 2021) due to consumer complaints about “perfume” issues with the products (discussed above in LAI). From August 1, 2014, through December 31, 2022, the overall reporting rate was 1.1 AE cases per one million Always HAI products shipped. For cases with known age group, 39.8% (*N* = 863) were reported in the older adult and 15.4% (*N* = 333) in adults (Table 5). Telephone (*N* = 1,463, 67.5%) and e-mail (*N* = 453, 20.9%) were the preferred reporting methods. The five most commonly reported PTs across all age groups were pruritis (skin, *N* = 386, 17.8%), rash (skin, *N* = 269, 12.4%), skin irritation (*N* = 256, 11.8%), genital discomfort (*N* = 224, 10.3%), and pain of skin (*N* = 193, 8.9%) (Table 6). The PTs for Always HAI correspond to expected AEs from HAI products that cover a broader region of skin including contacting the stomach, buttocks, and genitals. Each HAI case reported an average of 3.0 AEs (median = 2).

### 4.4. Advantages, limitations, and recommendations for post-marketing safety surveillance

This descriptive analysis assessed voluntary reports of AEs from Always AI products using a manufacturer's post-marketing surveillance system between August 1, 2014, and December 31, 2022. The AE profiles for Always LAI and MAI focused on symptoms in the genital area, and the Always HAI AE profile presented with both skin and genital AEs. Every AE (i.e., PT) across all three segments had a reporting frequency of less than 20%. Overall estimated reporting rates of AE cases combined from 2014 to 2022 were 0.3, 0.4, and 1.1 AE cases per one million Always AI products shipped for LAI, MAI, and HAI, respectively.

Benefits of having a post-marketing (passive) surveillance database include the ability to detect, investigate, and correct issues that occur after a product is introduced into the market (34). In the first half of 2022, a spike in cases occurred across all three AI segments. Consumers identified a stronger, different or new “perfume” smell after initially opening the AI product package and while handling or wearing the AI product. Quantitatively the “perfume” issue did not trigger a safety concern based on the frequency of PTs (Table 6). Complaints specifically describing the “perfume” as causing an AE were mapped to the PT “perfume sensitivity” (and to the corresponding AE PT including genital discomfort, asthma, and vulvovaginal rash). Other consumer complaints mentioning smell without specifying that the product's “perfume” was causing a health issue were only mapped to the AE PTs. By tracking and reading individual consumer comments qualitatively, the post-marketing surveillance AE data were supporting a product quality issue concerning “perfume” but not a safety issue. Therefore, the AE data along with other data sources within the company such as consumer, non-AE voluntary comments (data not shown) provided a more complete understanding of the potential “perfume” issue. As a result, the company took corrective action by reducing the amount of ONM in Always AI production during June of 2022. While the formula change did not impact the market immediately, quarterly AE cases received declined in all three segments from July through December of 2022 (data not shown).

There are several limitations in this descriptive analysis. Post-marketing (passive) surveillance depends on individuals to voluntarily report on the company's products, most likely representing only a fraction of all AE cases occurring in the population (i.e., selection bias). Incidence or prevalence cannot be calculated from these data since the actual number of AI products in use is unknown (i.e., lacking denominator data). Accordingly, we estimated reporting rates using shipments as a proxy. Analyses are further limited because statistical comparisons across segments should not be conducted with passive surveillance data that uses unsolicited voluntary reports. We assessed case counts across all geographies for each AI segment. It is recommended that when interpreting findings, one must include understanding of regional differences in product usage, shipment distribution (e.g., North America product distribution is two times Western Europe), voluntary reports, and vocality. Additionally, consumers may report complaints simultaneously on more than one product across segments (e.g., LAI and MAI). P&G's post-marketing surveillance system is designed at the individual consumer level (i.e., unit of analysis is a case) and not the product level. Hence, the PTs are counted for all products in the case, whether or not those symptoms (PTs) occurred with all products. Also, complete information is rarely provided by consumers (e.g., age group is unknown for over 40% of all cases in each segment, Table 5). Furthermore, potential reporting bias can result when external factors prompt voluntary reports (e.g., product coupons and social media).

Moreover, product or AE misclassification may occur as the self-reported data are not verified. Comments are coded per MedDRA terminology as described by consumers. An AE comment can be mapped to multiple PTs. Additionally, MedDRA's level of granularity can result in very specific PTs. For instance, events mentioning “itching” were mapped to the PT Pruritis, while those specifically stating “itchy—female genital” were mapped to the PT Pruritus genital. Coding differences can occur based on the specific words consumers use when reporting AEs (e.g., the “perfume” issue) and how these comments are then mapped per MedDRA terminology standards.

Post-marketing surveillance data are of utility to both regulatory bodies for monitoring devices and safety management for understanding long-term concerns. Regulatory compliance involves reviewing the data at the individual case level and assessing whether federal reporting is warranted for individual AE cases and aggregated case data according to country regulations. In addition to evaluation of individual cases, here we assessed safety at the population level by analyzing aggregate data on all cases. As shown, post-marketing surveillance data provide the ability to conduct descriptive research on AEs and disseminate evidence on product safety to a broader audience. It is recommended that other AI manufacturers publish their post-marketing surveillance findings in the literature to further develop the AE profiles for AI products.

Post-marketing surveillance provides ongoing monitoring of the long-term safety of consumer products. The findings from this descriptive analysis provide over eight years of data on the AE profiles for Always LAI, MAI, and HAI products, offering assurance that these products can be used safely.

## 5. Quality assurance processes

Medical device manufacturers are required by regulation to implement and maintain a robust quality assurance program. Quality improvement systems should be in place prior to manufacturing to ensure identification and control of suppliers, raw materials and packaging. The same quality criteria should be in place for a material regardless of the origin of that material. As AI products are considered class 1 medical devices in the EU, manufacturers are obliged by legislation to comply with numerous control processes and to maintain robust documentation of these processes. The STED file as previously discussed must include a description of the quality system and the respective supplier, raw material, manufacturing and finished product control processes.

The following paragraphs contain a summary of key aspects of the controls required by the medical device legislation. The controls outlined below ensure the expectations of ISO to assure the final product (e.g., as packaged over its lifetime) is representative of the product assessed in the biological evaluation plan.

The general quality approach that governs the manufacturing process for AI products includes the following elements which help assure safe use of the product:

• Every product component supplier is required to comply with GMP procedures at each site at which they produce. Suppliers must disclose detailed ingredients for their material and any subsequent changes to ensure they can be assessed for safety prior to use.
• Product components are selected according to strict quality criteria and during final manufacture/assembly, rigorous quality control systems and good manufacturing practices are in place to ensure the highest hygienic standards are met.
• Materials are rigorously qualified before use and tested frequently during normal production periods after qualification, including meeting requirements for potential impurities. These criteria are verified through analytical testing as appropriate.
• Extensive and rigorous controls are in place during the manufacturing process such as laboratory screening on selected product components, visual and subjective integrity, camera system defect detection, product control in internal and external laboratories. Similar rigor is required from each of the product component suppliers.
• All P&G products and manufacturing sites are regularly audited to ensure they consistently deliver the required product quality. For AI products, relevant external medical device standards include ISO 13485, FDA 21 CFR 820, Health Canada Standards and other applicable external regulatory standards where the product is manufactured or sold.
• In-market risk management programs including post–market surveillance and analysis of consumer experience, provide on-going data on product manufacturing and safety and can serve as an early-warning system to detect any unexpected, untoward effects.

## 6. Discussion/conclusions

The risk of an adverse effect from exposure to a chemical is dependent upon the inherent toxicity of the materials, the amount to which the consumer is exposed and the route of exposure. Based upon examination of the device materials and their individual constituents, use of the AI product would not be expected to result in exposure to chemicals at levels that would result in an unacceptable adverse biological response in consumers. The risk analysis was supported by information gathered from biological testing data on the material components (sensitization and irritation), published literature, and the long history of safe and effective use of the materials used to construct the product. This risk assessment indicates that the likelihood of a toxic effect from the incontinence products is negligible and that the device should be considered safe for use as intended. The evaluation of these AI products takes into consideration the exposure-based risk assessment results from the evaluation of each individual supplier disclosed chemical constituent, the extensive literature review of similar marketed products, clinical safety studies on these devices and closely related products, analytical data showing no impact on the extractable profile from the manufacturing process as well as years of favorable post-market safety surveillance data for these products. Taken together these data and approach described herein form a strong weight of evidence to support the safety and biocompatibility of the final, finished medical devices and compliance with regulatory requirements.
